# *mecC* MRSA in Israel—genomic analysis, prevalence and global perspective

**DOI:** 10.1093/jacamr/dlac085

**Published:** 2022-08-27

**Authors:** Moti Baum, Einav Anuka, Orit Treygerman, George Prajgrod, Lea Valinsky, Assaf Rokney

**Affiliations:** Public Health Laboratories–Jerusalem (PHL-J), Public Health Services (PHS), Ministry of Health (MOH), Jerusalem, Israel; Public Health Laboratories–Jerusalem (PHL-J), Public Health Services (PHS), Ministry of Health (MOH), Jerusalem, Israel; Central Laboratory, Meuhedet Health Services, Lod, Israel; Central Laboratory, Meuhedet Health Services, Lod, Israel; Public Health Laboratories–Jerusalem (PHL-J), Public Health Services (PHS), Ministry of Health (MOH), Jerusalem, Israel; Public Health Laboratories–Jerusalem (PHL-J), Public Health Services (PHS), Ministry of Health (MOH), Jerusalem, Israel

## Abstract

**Background:**

MRSA is a major global healthcare problem. In 2011, a new *mec* variant designated *mecC* was described, presenting partial identity at the DNA level, thus undetectable by routine *mecA* PCR.

**Objectives:**

Until now, no reliable information regarding *mecC* MRSA prevalence was available in Israel. In this study, to the best of our knowledge, we describe the first case of *mecC* MRSA in Israel, with focus on genomic analysis and global context.

**Methods:**

The *mecC* MRSA isolate was analysed by WGS with focus on phylogeny, global contextualization, virulence and resistance genes. The strain was characterized by antibiotic susceptibility testing, *spa* typing and presence of *mecA/C* and *pvl* genes.

**Results:**

An MRSA strain (SA10610), isolated from a urine sample of an 83-year old patient, was found negative for the *mecA* and *pvl* genes. The MLST and *spa* type were ST130 and t1736, respectively. SA10610 presented resistance to oxacillin, penicillin and cefoxitin, and susceptibility to all non-β-lactam agents tested. Phylogenetic comparison with a global dataset of 586 *mecC* MRSA genomes revealed substantial genomic divergence. The nearest genomic relatives were human and animal isolates from Denmark. A screen of 12 761 *S. aureus* isolates collected during 2011–18 in Israel indicated this is the only *mecC*-positive strain.

**Conclusions:**

A high degree of genetic variability was found between the SA10610 strain and previously sequenced *mecC* MRSA isolated worldwide. The genomic and phylogenetic analysis suggest that *mecC* MRSA isolates have evolved independently rather than from a common ancestor.

## Introduction

MRSA is a major bacterial human pathogen involved in a wide variety of diseases, ranging from relatively minor superficial skin infections to serious and life-threatening invasive infections. In addition to infections in humans, MRSA can cause diseases in a wide range of hosts including livestock, wildlife and companion animals.^1^

Resistance to β-lactams in *Staphylococcus aureus* is mediated by the *mec* genes, including *mecA* and *mecC*, which encodes an alternative PBP with a lower affinity for virtually all β-lactam antibiotics. The *mec* genes reside within a mobile genetic element named the staphylococcal cassette chromosome *mec* (SCC*mec*) and resistance to β-lactams is conferred by the acquisition of this cassette.^1^ Based on their genetic content and their structural organization, SCC*mec* elements have been classified into types and subtypes and to date 14 types (I–XIV) have been described.^2–4^

In 2011, during an epidemiological study of bovine mastitis, a new *mec* variant was described.^5^ This variant, named *mecC* (formerly *mecA*_LGA251_) exhibits only 70% nucleotide sequence homology with the classical *mecA* gene^6^ and 63% identity at the amino acid level.^5^ The new *mecC* gene is located on a novel SCC*mec* element, designated type –XI SCC*mec*. As a consequence of the limited *mecA*–*mecC* sequence homology and their respective proteins, *mecA* PCR and immunological tests targeting PBP2a fail to detect *mecC* MRSA, posing a diagnostic challenge for clinical microbiology laboratories.^5–7^

*S. aureus* can be found in the normal flora of healthy humans and animals. However, it can cause diseases in both hosts as an opportunistic pathogen. During the last few years a new type of MRSA has emerged, livestock-associated (LA) MRSA and infections with this type of MRSA have been increasingly reported worldwide especially in people with occupational livestock exposure.^8–11^ Clonal complex (CC) 130 is the most prevalent LA MRSA lineage in Europe although other lineages such as CC1 and CC7 also have been found to colonize and cause infections in livestock.

In the last decade, MRSA clones with a *mecC* gene have been detected in different animal species and humans, mainly in European countries but also on other continents,^12–18^ with isolates mainly belonging to CC130, CC1943 and CC425.^1,5,19^ Zoonotic transmission of *mecC* MRSA has been previously reported, although data on the prevalence, animal reservoir and epidemiology of *mecC* MRSA are still limited.^20,21^

This study represents, to the best of our knowledge, the first report of *mecC*-positive MRSA isolation in Israel, with focus on genomic and phenotypic characterization. Phylogeny and global context were analysed by genomic comparison with *mecC*-positive MRSA genomes isolated from humans and animals.

## Materials and methods

### Bacterial isolates, media and lysates

*S. aureus* SA10610 was isolated from a urine sample of an 83-year old male patient in October 2017. The sample had been submitted to the national *S. aureus* reference centre for bacteriological characterization. All MRSA and MSSA isolates from bacteraemia as well as MRSA from wound infections are referred to the national centre for further analysis. Between 2011 and 2018, 12 761 *S. aureus* isolates were analysed by in-depth strain characterization and typing. All isolates were stored in a deep freeze in our strain bank. *spa* types known to be prevalent in *mecC S. aureus* strains: t843, t1773, t978, t1535, t7189, t6293, t7947, t7485, t7946, t7734, t11702, t6220, t9280, t373, 528, t1048, t1532, t3218, t3256, t3570, t5970, t6300, t6292, t6386, t742, t11706, t978, t7945, t2345, t3391, t8835, t529. NCTC 13552 strain, *mecC* MRSA, was used as a control in *mecC* PCR reaction. Strain SA104 (*mecA* and *pvl* positive, *spa* type t008) was used as a positive control in *mecA*-PVL PCR and *spa* PCR. ATCC strain 43300 and 29213 were used as controls for Etest assay. All strains were cultured at 37°C for 16–24 h.

The strains were cultured on 4S agar^22^ and an isolate was transferred to Nutrient agar (PD040, hylabs, Israel) for further analysis. Lysis of bacterial cells was performed by suspending 1–2 colonies in 100 μL of lysis buffer (lysozyme 50.8 units; lysostaphin 2.7 units; TRIS 0.1 M pH = 8; EDTA 0.01 M; DDW to a final volume of 100 μL), incubation at 37°C for 30 min followed by boiling for 10 min. The lysate was centrifuged at 14000 rpm × 30 s and diluted 1:10, 1.5 μL of the diluted lysate was used as a template for PCR reaction.

### PCR

Multiplex PCR for the simultaneous detection of *mecA*, *pvl* and 16S rRNA gene, which serves as an internal amplification control, was performed as described by McClure *et al*.^23^ PCR for the detection of *mecC* gene was performed as described by Stegger *et al*.^24^ Verification of SCC*mec* type XI presence was performed as described by Garcia-Alvarez *et al*.^5^ PCR for the detection of immune evasion complex (IEC) genes, *chp* and *scn*, was performed as previously described.^25^ PCR for amplification of φ3 *int* was performed as described by Lekkerkerk *et al*.^26^

### Molecular typing

Molecular typing of the isolate by *spa* typing and MLST were performed as described previously.^27,28^*spa* typing and MLST analysis were performed using BioNumerics 7.6.3 software.

### Genomic DNA extraction, WGS analysis and bioinformatics analysis

Genomic DNA was extracted by using the GeneAid kit. DNA library was prepared using Nextera XT kit (Illumina Inc. San Diego, CA, USA). *De novo* assembly by SPAdes and whole genome MLST (wgMLST) analysis were performed on the BioNumerics 7.6.3 (Applied Maths, Belgium) using the default settings. Core genome MLST (cgMLST) comparison was performed by the BioNumerics software using 10 as scaling factor and categorical differences as similarity matrix.

The pubMLST *S. aureus* database was screened for *mecC*-positive isolates by BLAST. wgMLST schemes of *mecC*-positive strains were downloaded from pubMLST site, imported to the BioNumerics software and used for phylogeny analysis. Phylogeny was deduced by calculating a minimal spanning tree based on wgMLST allelic profiles.

Detection of resistance genes was carried out with the PATRIC tool using the Comprehensive Antimicrobial Resistance Database (CARD). Detection of virulence factors was carried out using the *S. aureus* functional genotyping tool.

### Antimicrobial susceptibility testing (AST)

All MRSA strains were grown at 37 ± 1°C for 18–24 h before conducting susceptibility tests. For Etest, several colonies were suspended in saline to a turbidity of 0.5 McFarland. The suspension was seeded on a Mueller–Hinton agar plate (Hy Laboratories Ltd, Rehovot, Israel) and then Etest strips (bioMérieux) were put on each agar plate for oxacillin and cefoxitin. Plates were incubated at 35 ± 1°C for 24 h (oxacillin) and 18–24 h (cefoxitin). MIC values were determined according to CLSI guidelines: resistance to oxacillin is defined at MIC values ≥4 mg/L; resistance to cefoxitin is defined at MIC values ≥8 mg/L.

Broth microdilution was performed with Sensititre susceptibility plates (Gram-positive GPALL1F AST Plate) according to manufacturer’s instructions. Briefly, ∼5 colonies were suspended in DDW to a turbidity of 0.5 McFarland. Then, 10 μL of the suspension were transferred into 11 mL of CAMHB (cat. Number T3462). The plate was inoculated using the Sensititre AutoInoculator/AIM. Following incubation for 24 h, results were read using the VIZION platform (Sensititre). MICs were determined according to CLSI guidelines (M100 2020).

Vitek AST was done with the Vitek 2 automated AST system using an AST-P649 card (bioMérieux, Marcy-l’Étoile, France).

### Data availability

The following genomes were used for WGS analysis comparison: *mec*A_LGA251_^5^ (accession: FR821779); M10/0061^7^ (accession: FR823292); Patient A^20^ (accession: ERR084771); Cow A^20^ (accession: ERR144792); Patient B^20^ (accession: ERR144788); Sheep B^20^ (accession: ERR144749); OFVD (accession: OFVD01000000); OFUF (accession: OFUF01000000); *mecC* MRSA isolated in Spain^29^ (accession: ERR403511); *mecC* MRSA isolated in Brazil^15^ (GCA_009763195.1); *mecC* MRSA isolated in New Zealand^29^ (ERR5417136); *mecC* MRSA isolated in England^29^ (ERR3595448); and *mecC* MRSA isolated in Australia^18^ (accession: LUFG00000000). WGS of strain SA10610 was deposited in the pubMLST database under the ID 34000.

## Results

Strain SA10610 was isolated from a urine sample from an 83-year old prostate carcinoma patient referred to the HMO due to a urinary tract infection. Vitek analysis (GP CARD) at the regional HMO laboratory identified the isolate as MRSA (positive cefoxitin screen and oxacillin MIC ≥4 mg/L), and the patient was treated with ofloxacin (Oflodex). In order to broaden the antibiotic resistance profile of the SA10610 isolate, broth microdilution testing was performed. SA10610 was tested for phenotypic susceptibility to common antimicrobial compounds indicating resistance to oxacillin, penicillin and cefoxitin and susceptibility to all other non-β-lactam agents tested (chloramphenicol, ciprofloxacin, levofloxacin, moxifloxacin, clindamycin, daptomycin, erythromycin, gentamicin, linezolid, rifampicin, tetracycline, trimethoprim/sulfamethoxazole, vancomycin and nitrofurantoin).

### Molecular characterization

Further analysis at the *S. aureus* national reference centre revealed that this strain is seemingly MSSA genotypically, negative for the *mecA* gene by PCR. The strain was phenotypically resistant to oxacillin and cefoxitin as determined by Etest (MIC values 24 mg/L and 32 mg/L, respectively), and Vitek 2 (0.5 mg/L and ≥6 mg/L, respectively). This observation was surprising and motivated us to check for the presence of the *mecC* gene. In PCR analysis for the *mecC* gene, SA10610 was found positive. The isolate was negative by PCR for the Panton–Valentine leukocidin (PVL) genes. MLST and *spa* typing assigned the isolate to ST130 and *spa* type t1736. PCR analysis for the detection of SCC*mec* type XI was positive.

We applied WGS in order to investigate the genomic context of the *mecC* gene, and the phylogenetic lineage of the strain. WGS of strain SA10610 revealed 100% identity to the *mecC* gene sequence of *S. aureus* strain TRN6234, isolated from a patient in a German hospital with a wound infection,^30^ and 99.9% identity to the *mecC* gene sequence of strain M10/0061 isolated from an 85-year-old male inpatient in a regional hospital in south Ireland in 2010.^7^ The SCC*mec* sequence of strain SA10610 showed 99.9% identity to SCC*mec* type XI of strain M10/0061.^7^

In addition, we compared the WGS-derived antimicrobial resistance profile of SA10610 isolate to that of the prototype *mecA*_LGA251_, along with genomes of *S. aureus* isolates isolated from human and animal origin worldwide. The results presented in Table 1 show agreement between all isolates tested except from *mecC* MRSA strain isolated from Sheep B and *mec*C MRSA isolated in Brazil, which were positive for pT181, a *tet*(K)-carrying plasmid, and Tn*552*, a β-lactamase-carrying transposon, respectively.

**Table 1.** WGS analysis for the presence of mobile genetic elements (MGE) conferring antibiotic resistance

Gene	Mobile genetic element	*S. aureus* isolate name										
		SA10610	mecA_LGA251_	Cow A	Patient A	Patient B	Sheep B	New Zealand	England	Spain	Australia	Brazil
*blaZ*	Tn*552*	−	−	−	−	−	−	−	−	−	−	+
*mecA*	SCC*mec*	−	−	−	−	−	−	−	−	−	−	−
*mecC*	SCC*mec*	+	+	+	+	+	+	+	+	+	+	+
*tet*(K)	pT181	−	−	−	−	−	+	−	−	−	−	−
*tet*(M)	Tn*916*	−	−	−	−	−	−	−	−	−	−	−

The genomes of the above isolates were tested using the PATRIC bioinformatics resource and BioNumerics for the presence of genes conferring antibiotic resistance.

We investigated the prevalence of *mecC* among a national strain bank maintained by the national reference centre. The fact that specific *spa* types were associated with *mecC-*positive *S. aureus* isolates motivated us to screen our strain bank database for common *spa* types, known to be prevalent in *mecC S. aureus* strains. None of the *mecC*-related *spa* types were found in our human-origin strains database. However, five *S. aureus* strains of animal origin (LSA25, LSA50, LSA52, LSA57 and LSA63) that belonged to *spa* type t529 were found in our database. Further analysis revealed that those strains are negative for *mecA*, *mecC* and *pvl* (Table 2).

**Table 2.** Data available for all strains analysed or mentioned in current article

Strain	Accession number	Source	*spa* type	ST	Gene presence					
					*mecA*	*mecC*	*pvl*	*scn*	*chp*	*ɸ3*
SA10610	34000 (pubMLST)	human	t1736	130	−	+	−	−	−	**+**
LSA25	NA	cattle	t529		−	−	−	−	−	**+**
LSA57	NA	cattle	t529		−	−	−	−	−	−
LSA63	NA	cattle	t529		−	−	−	+	−	**+**
LSA50	NA	cattle	t529		−	−	−	−	−	−
LSA52	NA	cattle	t529		−	−	−	−	−	−
mecA_LGA251_	FR821779 (NCBI)	milk container	t6300	425	−	+	−	−	−	NA
Patient A	ERR08477 (NCBI)	human	t843	130	−	+	−	−	−	NA
Cow A	ERR144792 (NCBI)	cattle	t843	130	−	+	−	−	−	NA
Patient B	ERR144788 (NCBI)	human	t843	130	−	+	−	−	−	NA
Sheep B	ERR144749 (NCBI)	sheep	t843	130	−	+	−	−	−	NA
Australia	LUFG00000000	cat	t6292	425	−	+	−	−	−	NA
Spain	ERR403511	deer	NA	425	−	+	−	−	−	NA
England	ERR3595448	hedgehog	t15289	6460	−	+	−	−	−	NA
New Zealand	ERR5417136	hedgehog	NA	6432	−	+	−	−	−	NA
Brazil	GCA_009763195.1	cow	t605	126	−	+^a^	−	−	−	NA

a *mecC* gene of this strain is not located in SCC*mec* type XI.

NA, not applicable.

### WGS analysis

**Figure 1.**
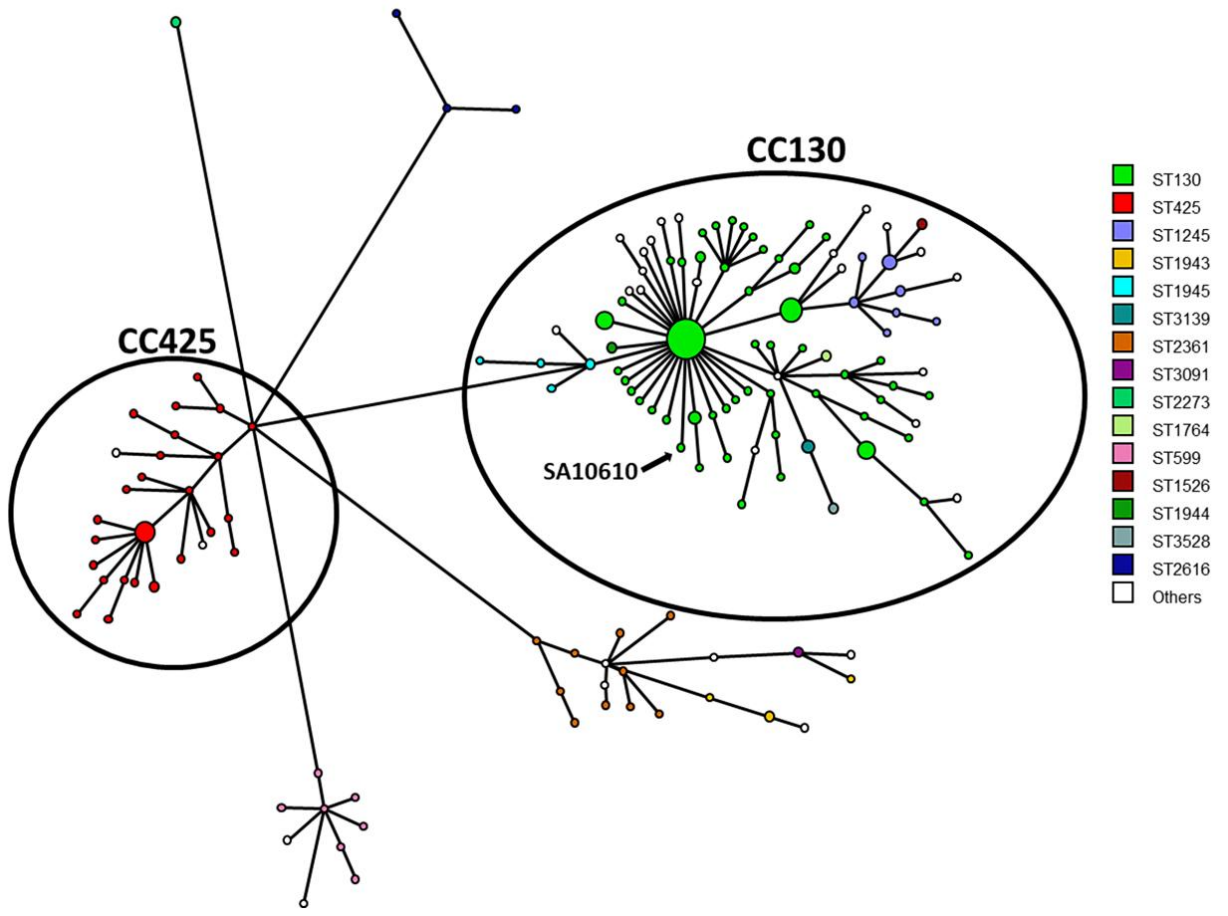
Minimum spanning tree of 586 *mecC* MRSA isolates based on wgMLST. Colours correspond to the STs of the samples. The distance between circles represents genetic divergence. Strains in which the distance is less than 120 alleles were combined into one node; circle size is proportional to the number of strains. Strain SA10610 is marked by arrow.

**Figure 2.**
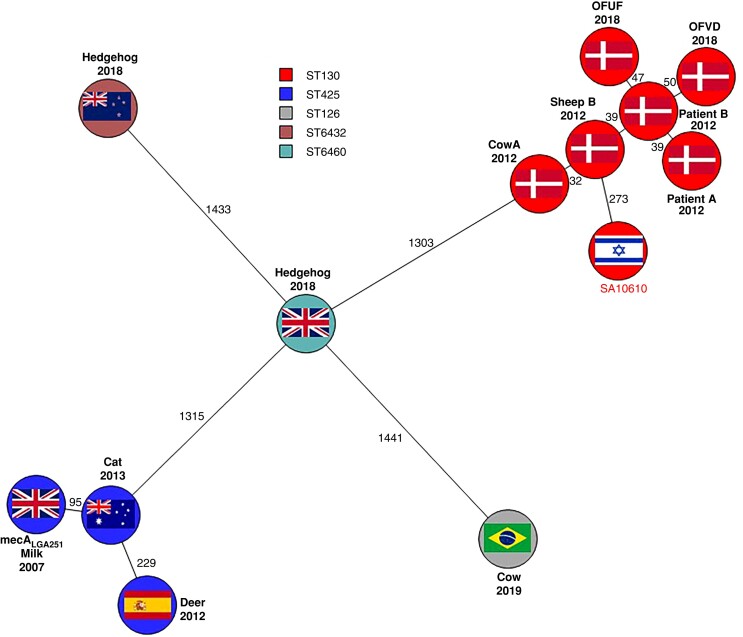
Phylogenetic relationships between SA10610 genome and other sequenced *mecC* MRSA genomes isolated worldwide. The figure shows a minimum spanning tree generated from wgMLST of sequenced *mecC* MRSA isolates generated by BioNumerics software. Each isolate is represented by a circle, the numbers of different alleles are shown in black. Flags inside the circle represents the country from where the isolates were isolated. The year of isolation is marked below the strain name.

*mecC* MRSA strains have been isolated from a vast range of countries and some of the genomes were deposited in public databases. We aimed to compare the SA10610 sequence to globally reported *mecC*-positive MRSA. Using the BioNumerics software, we generated a minimum spanning tree based on wgMLST data of 586 *mecC*-positive MRSA isolates whose genomic assemblies were deposited in the pubMLST database. The tree presented in Figure 1 shows that SA10610 is closely related to CC130. This large clade consists of 443 isolates that belong to CC130; of them, 327 belong to ST130 and the rest to closely related STs. We further assessed the diversity between SA10610 genome and the genome of the prototype *mecA*_LGA251_ along with other sequenced genomes of well-characterized *mecC* MRSA isolates by generating a minimum spanning tree on wgMLST data. The minimum spanning tree presented in Figure 2 shows that SA10610 is closest to isolates from Denmark and belongs to ST130. There is a clear distinction between SA10610 and *mecA*_LGA251_, *mecC* MRSA isolates isolated in Brazil, England and New Zealand. On the other hand, the genomes of strains Cow A, Patient A, Sheep B, Patient B, OFVD and OFUF clustered into one clade which is differentiated by 273 alleles from the SA10610 genome.

**Figure 3.**
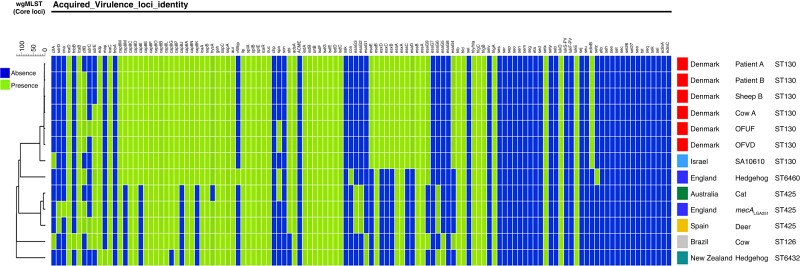
Comparison of virulence factors profile of SA10610 and other sequenced *mecC* MRSA genomes isolated worldwide. Virulence factors analysis was performed using the BioNumerics *S. aureus* functional genotyping tool. The blue/green colours represent absence/presence of genes, respectively. The dendrogram is based on cgMLST and was built using the BioNumerics software.

*mecC* MRSA isolates have been globally isolated from a wide range of diseases in humans and animals.^31^ We further investigated the virulence determinants and their relation to the cgMLST of the SA10610 isolate by analysis of the whole genome sequence, and compared it with *mecC* MRSA genomes including *mecA*_LGA251_ and *mecC* MRSA strains isolated worldwide.^20^ The results presented in Figure 3 show correlation between cgMLST and virulence profile analysis, the virulence profile of SA10610 is similar to CC130 strains. In addition, the similarity level of virulence profile decreases as the level of cgMLST variability increases.

### Livestock

The fact that *mecC* MRSA was first isolated from mastitis in a cow,^5^ and in addition that most of the patients from which *mecC* was isolated were in proximity to animals,^32^ stimulated us to try to determine its origin. In addition, the fact that five animal origin *S. aureus* strains of *spa* type t529, linked to CC130, were found in our database enabled us comparison between SA10610 isolate and animal origin strains. The results presented in Table 2 show that all isolates tested were negative for *scn*, *chp* and *ɸ3* PCR except from strains LSA25 and SA10610, which were positive for *ɸ3* only. LSA63 was positive for both *ɸ3* and *scn*. In addition these results are reinforced by the WGS analysis of the sequenced genome of strain SA10610, showing that strain SA10610 is negative for the human immune evasion genes *sak, chp* and *scn.*^33,34^ These genes are carried on the βC-ΦS bacteriophage alongside with *sea* genes, which encode the immune evasion molecule staphylokinase and enterotoxin A, respectively, which are also absent from the SA10610 genome.^25^ On the other hand, *tet*(M), an animal-origin marker, is also absent from the SA10610 genome.^33,34^

## Discussion

The first discovery of *mecC* MRSA was as a result of epidemiological study of bovine mastitis in 2007,^5^ subsequently, more *mecC*-positive isolates were identified, both of human and animal origin, in other countries. As far as we know, this is the first report of *mecC* MRSA in Israel, which is assigned *spa* type t1736 and ST130 and belongs to clonal complex CC130, which is the major lineage responsible for the vast majority of *mecC* isolates to date.^1^

The antibiotic susceptibility pattern of strain SA10610, as reported for most *mecC* MRSA isolates,^5,15,18,29,32^ is characterized by susceptibility to the majority of all non-β-lactam antibiotics.^35^ In addition, all the genomes tested in this analysis, except from the isolate isolated in Brazil, carry the same SCC*mec* and have very similar horizontally transmissible accessory genomes.

The patient from whom SA10610 was isolated lives in an urban environment and data regarding animal exposure was unavailable. Human-associated isolates carry phages encoding human innate immune modulators that are rare in livestock-associated isolates and therefore may be used as markers. These markers include the φSa3 phage, which contains an IEC of genes that include staphylococcal complement inhibitor (*scn*) and chemotaxis inhibitory protein (*chp*). Our comprehensive database, which incorporates data from 2011 onwards, was screened for the presence of *mecC*-harbouring MRSA isolates, based on *spa* types that are characteristics of *mecC*-positive *S. aureus*. Five MSSA isolates of animal origin were found. Those isolates were negative for *chp*, four were negative for *scn* and two were positive for *φSa3*. In addition, WGS sequences of *mecC* MRSA genomes isolated worldwide from humans and animals were screened for the presence of *scn* and *chp* genes and were found negative. In light of the above, it is hard to identify the cause or source of the infection.

*mecC* MRSA is considered as animal-adapted lineages that apparently emerged in animals and may later spread to humans.^20,21,29^ In spite of the fact that it was detected in a single patient out of our entire database, the isolation of a *mecC*-positive strain is a fact of supreme importance given the increase in prevalence of *mecC* MRSA reported worldwide and due to its public health relevance and its zoonotic potential.

After the original discovery of *mecC* MRSA in the UK, more cases were reported, most of them belonged to CC130 as determined by MLST.^36^ In this report we are trying to consider our findings in a broader perspective by comparing the WGS results of *mecC* MRSA isolates worldwide to SA10610 isolates. Our analysis outlines the genomic divergence between strains that belongs to different STs. This distinction is also reinforced by, firstly, the difference in virulence factor profiles: SA10610 is closer in its virulence arsenal to the genomes isolated in Denmark, while *mecA*_LGA251_ and the Australian strain are similar one to another but considerably different from the rest of the tested strains. It is worth mentioning that there is a slight difference between the SA10610 isolate and the ‘Denmark clade’. The distance between SA10610 isolate and the Denmark clade is 273 alleles, a distance significantly greater than the distances between the other isolates belong to the Denmark clade. Additionally, while SA10610 is of *spa* type t1736, the strains that belong to the Denmark clade are of *spa* type t843. Secondly, the dendrogram based on cgMLST, presented in Figure 3 divides the tested strains into several groups that are well differentiated from each other. This division is in alignment with the clustering into STs and CCs. Considering the wider context, *mecC* MRSA isolates are associated mainly with CC130, but also with a wide variety of other STs.^29^ The fact that the distance between the clades is substantially high (∼1500 alleles) may imply the evolutionary origins of *mecC* MRSA. It seems that there is no one ancestral *mecC* MRSA origin from which descendant isolates evolved. Rather, *mecC* MRSA emerged in different places independently of each other.

In conclusion, as far as we know, this study represents the first report of *mecC* MRSA in Israel. The genomic and epidemiologic information presented in this research can support further *mecC* MRSA studies. The relative similarity between the SA10610 strain and previously sequenced *mecC* MRSA CC130 isolated from humans and animals in Denmark, in the cgMLST, resistance and virulence profiles may reflect a weakly clonal lineage.
